# Variability in detection of SARS-CoV-2-specific antibody responses following mild infection: a prospective multicentre cross-sectional study, London, United Kingdom, 17 April to 17 July 2020

**DOI:** 10.2807/1560-7917.ES.2022.27.4.2002076

**Published:** 2022-01-27

**Authors:** Scott JC Pallett, Rachael Jones, Ahmed Abdulaal, Mitchell A Pallett, Michael Rayment, Aatish Patel, Sarah J Denny, Nabeela Mughal, Maryam Khan, Carolina Rosadas de Oliveira, Panagiotis Pantelidis, Paul Randell, Christofer Toumazou, Matthew K O’Shea, Richard Tedder, Myra O McClure, Gary W Davies, Luke SP Moore

**Affiliations:** 1 Centre of Defence Pathology, Royal Centre for Defence Medicine, Queen Elizabeth Hospital Birmingham, Birmingham, United Kingdom; 2 Chelsea and Westminster Hospital NHS Foundation Trust, London, United Kingdom; 3 Department of Infectious Disease, MRC Centre for Molecular Bacteriology and Infection, Imperial College London, United Kingdom; 4 North West London Pathology, London, United Kingdom; 5 Department of Infectious Disease, Faculty of Medicine, Imperial College London, St Mary’s Campus, London, United Kingdom; 6 Faculty of Engineering, Department of Electrical and Electronic Engineering, Imperial College London, South Kensington Campus, London, United Kingdom; 7 Institute of Immunology and Immunotherapy, College of Medical & Dental Sciences, University of Birmingham, Edgbaston, Birmingham, United Kingdom; 8 Imperial College London, NIHR Health Protection Research Unit in Healthcare Associated Infections and Antimicrobial Resistance, London, United Kingdom

**Keywords:** COVID-19, Diagnostics, Coronavirus

## Abstract

**Introduction:**

Immunoassays targeting different SARS-CoV-2-specific antibodies are employed for seroprevalence studies. The degree of variability between immunoassays targeting anti-nucleocapsid (anti-NP; the majority) vs the potentially neutralising anti-spike antibodies (including anti-receptor-binding domain; anti-RBD), particularly in mild or asymptomatic disease, remains unclear.

**Aims:**

We aimed to explore variability in anti-NP and anti-RBD antibody detectability following mild symptomatic or asymptomatic SARS-CoV-2 infection and analyse antibody response for correlation with symptomatology.

**Methods:**

A multicentre prospective cross-sectional study was undertaken (April–July 2020). Paired serum samples were tested for anti-NP and anti-RBD IgG antibodies and reactivity expressed as binding ratios (BR). Multivariate linear regression was performed analysing age, sex, time since onset, symptomatology, anti-NP and anti-RBD antibody BR.

**Results:**

We included 906 adults. Antibody results (793/906; 87.5%; 95% confidence interval: 85.2–89.6) and BR strongly correlated (ρ = 0.75). PCR-confirmed cases were more frequently identified by anti-RBD (129/130) than anti-NP (123/130). Anti-RBD testing identified 83 of 325 (25.5%) cases otherwise reported as negative for anti-NP. Anti-NP presence (+1.75/unit increase; p < 0.001), fever (≥ 38°C; +1.81; p < 0.001) or anosmia (+1.91; p < 0.001) were significantly associated with increased anti-RBD BR. Age (p = 0.85), sex (p = 0.28) and cough (p = 0.35) were not. When time since symptom onset was considered, we did not observe a significant change in anti-RBD BR (p = 0.95) but did note decreasing anti-NP BR (p < 0.001).

**Conclusion:**

SARS-CoV-2 anti-RBD IgG showed significant correlation with anti-NP IgG for absolute seroconversion and BR. Higher BR were seen in symptomatic individuals, particularly those with fever. Inter-assay variability (12.5%) was evident and raises considerations for optimising seroprevalence testing strategies/studies.

## Introduction

Severe acute respiratory syndrome coronavirus-2 (SARS-CoV-2) emerged as a novel respiratory pathogen in late 2019. Having spread extensively, coronavirus disease (COVID-19) was declared a global pandemic on 11 March 2020 [1]. Case finding strategies during the acute phase of infection have principally relied on detection of viral RNA by PCR [2]. Prevalence estimates have been further complicated by asymptomatic infection [3]. Serological assays have since been implemented in delayed case identification programmes (identification of previously asymptomatic or symptomatic individuals with negative PCR or untested cases of infection) for high-risk populations [4], and in some cases as part of national serosurveillance studies, in order to better estimate disease prevalence [5].

While characterisation of the immune response to infection with SARS-CoV-2 is ongoing, it appears that the initial humoral response occurs before the recovery phase. Detection of SARS-CoV-2-specific antibodies are observed early in the convalescent period, and appear to persist for at least 5–6 months [6-8]. While many commercial assays detect antibodies to the highly conserved virus nucleocapsid (NP) [9], it is antibodies to the spike protein, and specifically the receptor-binding domain (RBD) with its role in virus attachment and cell entry, where a correlation has been made with neutralisation [10]. Antibody detectability has broadly been associated with disease severity [11]. Inter-assay agreeability however remains unclear, particularly following mild or asymptomatic infection [12]. If significant inter-assay variability exists, there may be implications for long-term serological studies through under-reporting of infected cases. Additionally, as new treatment options such as combination monoclonal antibody therapies have shown benefit in prevention and treatment of severe COVID-19 only if individuals are anti-spike antibody seronegative at baseline, any inter-assay variability will need to be understood in order to interpret results correctly [13].

Investigation of differential SARS-CoV-2 antibody responses and potential variability between anti-NP and anti-RBD immunoassays could allow further optimisation of testing strategies. In this study, we explore the dose–response relationship of an in-house anti-RBD IgG assay and an anti-NP IgG assay employed by the United Kingdom (UK) Government to support testing in the National Health Service.

The aim of this study was to explore potential variability between a commercial anti-NP and the in-house anti-RBD antibody immunoassay in high-risk populations and assess whether a dose–response relationship is apparent following mild symptomatic or asymptomatic SARS-CoV-2 infection. The main objective was to explore variability between anti-RBD antibody assay performance and a current anti-NP assay standard by measuring sensitivity against PCR-positive cases and concordance between anti-NP and anti-RBD antibody detection across the entire cohort. Secondary objectives were to evaluate relationships between antibody binding ratios (BR, both anti-RBD and anti-NP), participant demographics and severity of symptoms, and to evaluate relationships between antibody BR (both anti-RBD and anti-NP) and time since symptom onset in symptomatic cases.

## Methods

### Study setting and design

A prospective multi-centre programme for serological identification of SARS-CoV-2 cases in the community was implemented from 17 April 2020 to 17 July 2020 in London, UK. Participants were invited from a delayed case identification programme developed in response to a national antibody testing initiative [14]. As part of this initiative, the programme was responsible for offering antibody testing to all healthcare workers (HCW) at two separate hospitals as well as to all HCW and residents at long-term care facilities (LTCF) local to Chelsea and Westminster Hospital on a voluntary basis. The programme offered serological testing on whole blood by chemiluminescent microparticle immunoassay (CMIA; Abbott IgG anti-nucleocapsid CMIA; Abbott Laboratories, Lake Bluff, United States (US)) [4]. For those individuals who consented to give a blood sample for anti-NP CMIA testing, consent was also sought for a second sample to be sent for paired anti-RBD testing. All symptomatic patients in this programme had been managed in the community, with severe cases (requiring hospitalisation) excluded, otherwise no limitation was set on eligibility in terms of specific symptom or exposure risk; invitation was extended equally to participants from both HCW and LCTF cohorts in order to provide results across a wide spectrum of age.

All participants underwent screening by clinical staff who collected a history of symptoms and clinical history, performed venepuncture and counselled the participants on the results. If previously symptomatic, based on a case definition of new onset of cough, fever (≥ 38 °C), breathlessness or anosmia with onset after the date of the first confirmed local case (West London, 27 February 2020), we collected data on the time of initial symptom onset. Samples were collected more than 14 days after symptom onset and after the participant had recovered from fever and cough. All samples were provided with anonymous identifiers before testing.

### Anti-nucleocapsid antibody testing

As per the local clinical testing algorithm, serology for consenting participants was initially conducted using the Abbott CMIA targeting SARS-CoV-2-specific anti-NP IgG [15]. Operators of the immunoassay were blind to previous PCR results, symptom duration and severity. Results were reported as a calculated BR, derived by dividing sample chemiluminescent signal by a cut-off signal established by the manufacturer ARCHITECT *i* System from three negative controls [16]. Results are reported as either not detected (BR < 1.39) or detected (BR ≥ 1.40) as recommended by the manufacturer. Public Health England have previously evaluated the Abbott IgG CMIA and report a sensitivity of 93.5% (95% confidence interval (CI): 85.5–97.9) and specificity of 100.00% (95% CI: 99.1–100.0) [16]. Our previous work reported a sensitivity of 87.5% (95% CI: 75.3–92.9) and a specificity of 95.0% (88.72–98.36%) for this assay [4].

### Anti-receptor-binding domain antibody testing

Samples were tested using the Imperial hybrid double antigen binding assay (DABA) for detection of anti-RBD IgG (Imperial College London, London, United Kingdom), a two-step sequential enzyme linked immunoassay which utilises the S1 subunit of the spike protein on the solid phase and labelled RBD in the fluid phase. Previous analysis has shown this assay to be 100% (95% CI: 99.6–100) specific, defined by testing 825 sera that pre-dated the COVID-19 pandemic, and 98.9% (95% CI: 96.8–99.8) sensitive when evaluating 276 sera from RT-PCR-confirmed individuals [12]. Operators of the assay were blind to previous PCR results, symptom duration and severity. Assay reactions were read spectrometrically (450–630 nm) and a cut-off was derived by adding 0.1 to the average optical density in three negative controls included in each run [17]. A BR was subsequently established for each sample by dividing the individual sample OD by the cut-off value. A sample giving a BR ≥ 1.0 was considered to contain detectable anti-RBD and correlated with neutralising activity. Pre-pandemic sera (collected before June 2019) were used for specificity testing and included (i) 94 samples from blood donors provided by NHS Blood Transfusion, Scotland, (ii) 498 samples from the Airwaves study [18], (iii) 100 samples from antenatal screening and (iv) 133 samples from patients with human T-lymphotropic virus type-1 infection. All these samples tested negative. Full details of specificity testing, methodology and cut-off calculations for the hybrid DABA are available [17].

### Statistical analysis

We employed a pre-specified statistics plan. Descriptive statistics were used to analyse participant demographics, clinical symptoms, PCR results and paired antibody assays. Where available, serological results of both assays were assessed for their ability to detect prior PCR-confirmed infection. Concordance between the anti-NP and anti-RBD antibody test results were reported. Paired data were compared using McNemar’s test. The relationship between matched paired results and discordant results (i.e. only one assay positive) were initially assessed with the Mann–Whitney U test for BR and for time since symptom onset. To test unadjusted correlation between anti-NP and anti-RBD BR, we used Spearman’s rank correlation coefficient testing, as these variables were not normally distributed following Shapiro–Wilk testing. Continuous variables were normalised using a two-step transformation [19]. Time since symptom recovery was initially compared for both anti-NP and anti-RBD using the Kruskal–Wallis test and Dunn’s post-hoc pairwise comparisons for week-by-week data conducted for significant results. In order to assess any relative impact on the degree of positivity of anti-RBD BR, we then analysed the variables in a multivariate linear regression model. The model included age, sex, anti-NP BR and individual symptoms (fever, cough and/or breathlessness, anosmia [2]) to assess their respective correlations with anti-RBD BR across the entire cohort. We repeated the model for a sub-group analysis of symptomatic participants with the addition of time since symptom onset to the other tested variables. The significance threshold was set to p < 0.050. All statistical analyses were conducted using IBM SPSS Statistics (Version 26.0; IBM Corportation, Armonk, NY, US).

### Ethical statement

The study was a service evaluation of a delayed case identification programme authorised by the Chelsea and Westminster NHS Foundation Trust COVID-19 Gold Strategic Board (8 April 2020). Participants gave consent for paired anti-NP and anti-RBD antibody testing of serum samples. Where individual cases are discussed, written informed consent was obtained. Residual sera from historic samples were used as per UK Standards for Microbiology Investigations (Public Health England gateway number: 2015306) and in accordance with The Use of Human Organs and Tissues Act 2004, where ethical approval is not required for the use of residual sera in kit validation or evaluation.

## Results

A total of 906 individuals (652 HCW, 254 LTCF participants) underwent paired anti-NP and anti-RBD antibody testing between 17 April and 17 July 2020. The overall median age was 47 years (interquartile range (IQR): 39 years) and 471 of 902 (52.2%) were female. Median age for HCW was 37 years (IQR: 24 years) and for LTCF residents 78 years; IQR: 16 years). Participants were included based on consecutive consenting individuals for paired testing, and all 906 participants were included in the analysis. No screened individuals were excluded and no assay failures were recorded. Of those tested, 130 had previously been confirmed to have SARS-CoV-2 infection by PCR at least 14 days before sampling for serological testing.

### Anti-NP and anti-RBD seroconversion

A total of 581 of 906 (64.1%) individuals were seropositive for anti-NP IgG (median serum BR: 3.78; IQR: 4.47) and 325 (35.9%) were seronegative for anti-NP (median serum BR: 0.07; IQR: 0.47). In contrast, 634 of 906 (70.0%) individuals were seropositive for anti-RBD (median BR: 10.0; IQR: 13.5) and 272 (30.0%) were seronegative for anti-RBD (median BR: 0.25; IQR: 0.14). There was a significant difference in anti-NP and anti-RBD seropositivity (McNemar’s chi-square value = 24.9; p < 0.001). Concordance across both assays for paired samples with either a negative or positive result was seen in 793 of 906 (87.5%; 95% CI: 85.2–89.6 (Table 1)). An increase in detection rate through anti-RBD antibody testing of anti-NP antibody-negative results demonstrated a comparative positive pick-up rate of an additional 83 of 906 (9.20%; 95% CI: 7.4–11.2); Table 1). There was a significant difference in time since symptom onset between those who had a discordant result (i.e. paired results positive for one assay only) and those with matched results (63.8 days (IQR: 55.2) for mis-matched results and 42.6 days (IQR: 50.8) for matched results; p = 0.005). In anti-NP antibody-positive, anti-RBD antibody-negative samples, anti-NP BR were significantly lower than in samples with matched results (p < 0.001); the same was true for BR in anti-NP antibody-negative, anti-RBD antibody-positive samples (p = 0.005).

**Table 1:** Sensitivity of the SARS-CoV-2 in-house anti-RBD assay and Abbott anti-NP assay and correlation of results across the entire cohort, London, United Kingdom, 17 April–17 July 2020 (n = 906)

		Anti-RBD-positive	Anti-RBD-negative	Sensitivity % (95% CI)	Concordance % (95% CI)	Days since symptom onset Median (IQR)	Binding ratio Median (IQR)
PCR-positive cases (n = 130)	Anti-NP positive	123	0	Anti-RBD: 99.2 (95.8–100.0) Anti-NP: 94.6 (89.2–97.8)	95.4 (90.2–98.3)	36 (54)	Anti-RBD: 15.3 (12.7) Anti-NP: 6.42 (4.45)
	Anti-NP-negative	6	1				
Symptomatic cases (n = 521)	Anti-NP positive	421	14	NA	88.5 (85.4–91.1)	46.5 (50)^a^	Anti-RBD 9.55 (14.2) Anti-NP: 3.98 (4.67)
	Anti-NP-negative	46	40				
Asymptomatic cases (n = 385)	Anti-NP positive	130	16	NA	86.2 (82.4–89.5)	NA	Anti-RBD: 0.46 (5.27) Anti-NP: 0.39 (2.16)
	Anti-NP-negative	37	202				

anti-NP: anti-nucleocapsid antibody; anti-RBD: anti-receptor binding domain antibody; BR: binding ratio; CI: confidence interval; IQR: interquartile range; NA: not applicable; SARS-CoV-2: severe acute respiratory syndrome coronavirus-2.

^a^ For time since symptom onset for symptomatic cases, data represent those with clearly defined date of onset (n = 376).

Calculated sensitivities of both the in-house anti-RBD and the Abbott anti-NP assay with comparison against SARS-CoV-2 PCR-positive cases. Agreement between assay results (positive and negative) are presented with a breakdown for both symptomatic and asymptomatic cases.

### Anti-NP and anti-RBD seroconversion among PCR confirmed participants

In total 203 of 521 symptomatic participants underwent testing with a nasopharyngeal swab for acute phase PCR, and 130 of them were positive. The remainder had symptoms before widespread availability of PCR testing for HCW and were not tested during their acute symptomatic phase. Among the 130 participants with a PCR-confirmed infection, 123 (sensitivity: 94.6%; 95% CI: 89.2–97.8) demonstrated a positive anti-NP result and 129 (sensitivity: 99.2%; 95% CI: 95.8–100.0) a positive anti-RBD result. There was a significant difference in anti-NP and anti-RBD seropositivity (McNemer’s chi-square value = 6.00, p = 0.014). Concordance between the two serological assays among PCR-positive participants was 95.4% (95% CI: 90.2–98.3) (Table 1).

### Disease severity correlates with differential antibody response

**Figure 1:**
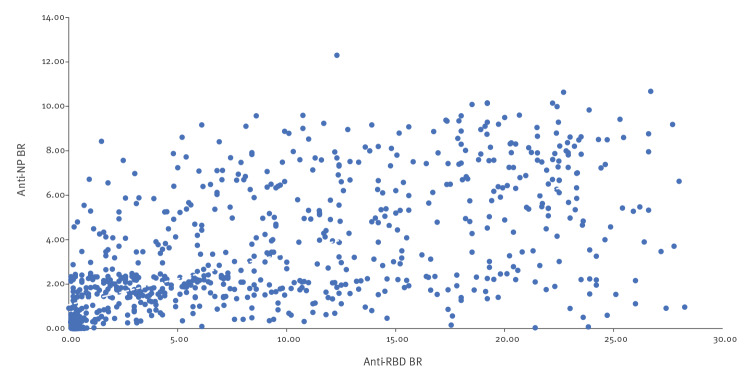
Correlation between SARS-CoV-2 anti-NP and anti-RBD antibody response London, United Kingdom, 17 April–17 July 2020 (n = 906)

Shapiro–Wilk testing (for BR of anti-NP and anti-RBD) showed that both variables were not normally distributed. Spearman’s rank correlation coefficient demonstrated a strong correlation between BR (ρ = 0.75; p < 0.0001; Figure 1).

anti-NP: anti-nucleocapsid antibody; anti-RBD: anti-receptor binding domain antibody; BR: binding ratio; SARS-CoV-2: severe acute respiratory syndrome coronavirus-2.

Graphical representation of Spearman’s rank correlation for anti-NP (chemiluminescent microparticle immunoassay, Abbott Laboratories, Lake Bluff, United States) and anti-RBD (double antigen binding assay, London, United Kingdom) BR for 906 paired samples from asymptomatic and symptomatic patients presenting for SARS-CoV-2 serological testing.

**Figure 2:**
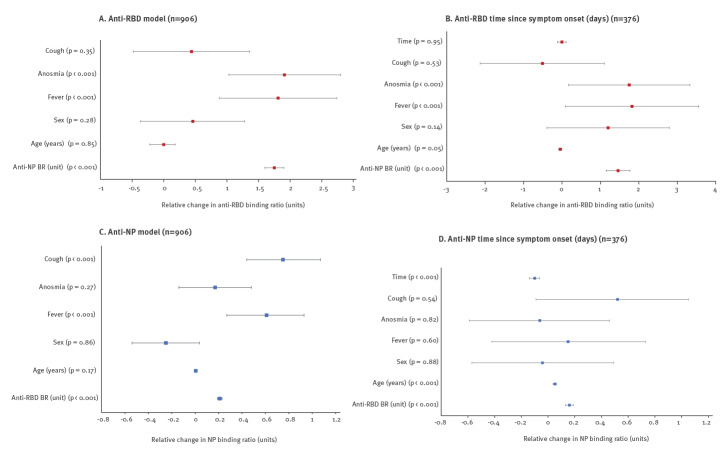
Multivariate linear regression model of clinical variables associated with SARS-CoV-2 anti-RBD antibody response, London, United Kingdom, 17 April–17 July 2020 (n = 906)

Following normalisation of continuous variables, a multiple linear regression model including all patients was run for both anti-RBD and anti-NP. The anti-RBD model had an R^2^ = 0.56 and the anti-NP model an R^2^ = 0.52. In the anti-RBD BR model, the presence of fever or anosmia was significantly positively associated with the anti-RBD BR value, which increased in the presence of fever by 1.81 (95% CI: 0.88–2.74; p < 0.001) and for anosmia by 1.91 (95% CI: 1.03–2.80; p = 0.001) (Figure 2A, Table 2). Anti-NP BR was also significantly associated with anti-RBD BR values (anti-RBD BR increasing by 1.75; 95% CI: 1.60–1.90; p < 0.001 for each anti-NP BR unit increase). The model found age to be negatively associated with the anti-RBD BR result, which decreased by 0.002 (95% CI: −0.22 to 0.018; p = 0.085) for each year of increased age (Figure 2A, Table 2). There was no significant association demonstrated with cough (p = 0.35) or sex (p = 0.28). The anti-NP BR model also found a significant association with fever, increasing the BR value by 0.61 (95% CI: 0.27–0.93; p < 0.001). In contrast, it found a significant association with cough, increasing the BR value by 0.75 (95% CI: 0.44–1.07; p = 0.01) when present, and it did not find a significant association with anosmia (p = 0.27) (Figure 2C, Table 2). Anti-RBD BR was significantly associated with an increased anti-NP BR (increase of 0.21 (95% CI: 0.19–0.23; p < 0.001) for each unit increase of anti-RBD BR). Neither age (p = 0.17) nor sex (p = 0.87) were associated with a significant change in anti-NP BR.

anti-NP: anti-nucleocapsid antibody; anti-RBD: anti-receptor binding domain antibody; BR: binding ratio; SARS-CoV-2: severe acute respiratory syndrome coronavirus-2.

All 906 participants are included in the analysis in panels A and C; Panel B (anti-RBD BR model) and panel D (anti-NP BR model) include 376 participants (symptomatic individuals with clear date of symptom onset during the study period) in the analyses of time since symptom onset . All participants had a documented history of symptoms or documentation of no history of symptoms available. Among the 521 cases with symptoms, fever was reported in 346 (66.4%), cough in 334 (64.1%) and anosmia in 202 (38.8%). Relative change in anti-RBD BR is expressed per 1 BR unit. The results for fever (> 38 °C), anosmia and cough show relative increase or decrease in anti-RBD BR or anti-NP BR based on presence of that symptom. Age represents relative change in BR per year increase in age. Anti-NP BR represents the relative anti-RBD BR increase based on each single unit increase of anti-NP BR (panels A and B) and vice versa for panels C and D. Sex shows relative change in BR associated with female sex. Time is representative of relative change in BR per day between serological sampling and day 14 of symptoms. Whiskers represent lower and upper 95% confidence intervals.

**Table 2:** Multivariable analysis of potential factors associated with anti-NP and anti-RBD BR values for SARS-CoV-2, London, United Kingdom, 17 April–17 July 2020 (n = 906)

Category	Dependent	Variable	Relative change in BR value	95% CI	p value
Anti-RBD model (n = 906)	Anti-RBD BR	Anti-NP BR (per change of 1 unit)	1.75	1.60 to 1.90	< 0.001
		Age difference (years)	−0.002	−0.22 to 0.18	0.85
		Sex	0.46	−0.37 to 1.28	0.28
		Fever	1.81	0.88 to 2.74	< 0.001
		Anosmia	1.91	1.03 to 2.80	< 0.001
		Cough	0.44	−0.48 to 1.36	0.35
Anti-RBD: days since symptom onset (n = 376)	Anti-RBD BR	Anti-NP BR (per change of 1 unit)	1.46	1.15 to 1.77	< 0.001
		Age difference (years)	−0.04	−0.09 to −0.01	0.047
		Sex	1.20	-0.39 to 2.79	0.14
		Fever	1.82	0.09 to 3.56	< 0.001
		Anosmia	1.75	0.17 to 3.33	< 0.001
		Cough	−0.51	−2.13 to 1.10	0.53
		Time	−0.003	−0.12 to 0.11	0.95
Anti-NP model (n = 906)	Anti-NP BR	Anti-RBD BR (per change of 1 unit)	0.21	0.19 to 0.23	< 0.001
		Age difference (years)	0.005	−0.002 to 0.01	0.17
		Sex	−0.25	−0.54 to 0.04	0.86
		Fever	0.61	0.27 to 0.93	< 0.001
		Anosmia	0.17	−0.14 to 0.48	0.27
		Cough	0.75	0.44 to 1.07	< 0.01
Anti-NP: days since symptom onset (n = 376)	Anti-NP BR	Anti-RBD BR (per change of 1 unit)	0.16	0.13 to 0.19	< 0.001
		Age difference (years)	0.05	0.04 to 0.06	< 0.001
		Sex	−0.04	−0.57 to 0.49	0.88
		Fever	0.15	−0.42 to 0.73	0.60
		Anosmia	−0.06	−0.59 to 0.46	0.82
		Cough	0.52	−0.09 to 1.05	0.54
		Time	−0.10	−0.14 to −0.07	< 0.001

anti-NP: anti-nucleocapsid antibody; anti-RBD: anti-receptor binding domain antibody; BR: binding ratio; CI: confidence interval; SARS-CoV-2: severe acute respiratory syndrome coronavirus-2.

Reported data from paired serological testing of asymptomatic (385/906) and symptomatic (521/906) patients presenting for SARS-CoV-2 serological testing. Relationship to anti-RBD BR and anti-NP BR and significance is presented for all variables included in the multiple linear regression model (age, sex, individual symptoms (fever, cough, anosmia) and paired antibody BR value as possible predictor variables for the dependent antibody BR variable. The model is repeated for the 376 individuals with clear onset date of symptoms meeting the case definition in order to assess any correlation with time since symptom onset during our study period.

### Time since symptom onset

Of 906 participants, 521 (57.5%) had a history of symptoms that matched the study case definition for SARS-CoV-2 infection [2]. The remaining, asymptomatic, individuals were excluded from exploring temporal associations. Among these 521 participants, 376 (72.2%) had symptom onset dates associated with a clear, identifiable, single bout of symptoms. The remaining participants (145/521; 27.8.%) reported multiple episodes of symptoms matching the case definition before serological testing or were unable to accurately recall symptom onset and so were removed from time–BR relationship analysis. Among the 376 assessed for temporal associations, the median time since symptom onset was 46.5 days (IQR: 50). Grouped into weeks to allow sufficient comparison across the cohort, Kruskall–Wallis test for variation in anti-RBD BR against time since symptom onset showed very strong evidence of a difference in BR between individual weeks since symptom onset for anti-RBD and for anti-NP (p = 0.004 and p < 0.001, respectively) (Supplementary Figure S1 is a visualisation of change in antibody BR on a week-by-week basis from symptom onset). Dunn’s pairwise comparisons are provided (Supplementary Figure S2 compares each antibody BR from each individual week with each of the other weeks within the time period). Repeating the multivariable regression model with the inclusion of time since symptom onset re-produced similar findings for significance for the anti-RBD model with the addition of a non-significant association of anti-RBD BR value when considered against time since symptom onset for our tested duration (Figure 2B, Table 2). Conversely, the anti-NP BR model showed a change to a small but significant association with age (increase of 0.051 for each year; 95% CI: 0.037–0.064; p < 0.001) and a significant association with decreasing anti-NP BR against time since symptom onset itself, decreasing by 0.10 (95% CI: −0.14 to −0.065; p < 0.001) for each day beyond 14 days since symptom onset (Figure 2D, Table 2).

### Initial presentation of cases requiring further clinical interpretation

A single case of SARS-CoV-2 infection confirmed by PCR was found to be negative in both anti-NP and anti-RBD assays.

This case was a man in his 90s with a new onset cough and fever with an oxygen requirement, chest radiograph changes suggestive of SARS-CoV-2 infection and prolonged contact with a known positive case of SARS-CoV-2 infection. A SARS-CoV-2 PCR (AusDiagnostics, Syndney, Australia; second stage Cq 16.05) was positive on 22 March 2020. Serological sampling undertaken on 20 April, 15 May and 18 June 2020 were negative for anti-NP and anti-RBD. At the time of presentation, an additional nasopharyngeal swab was sent for PCR testing for influenza A and B as well as respiratory syncytial virus, all of which were negative.

### Investigation of cases requiring further clinical interpretation

Resampling was undertaken on 23 July 2020 and showed an anti-RBD BR of 0.20. Further investigation on the same day showed a lymphocyte count of 1.6 × 10^9^/L (0.9–1.6 × 10^9^/L over the preceding 83 days), with lymphocyte subsets demonstrating a low relative B-cell count (45 cells/μL, normal range: 100–500, B lymphocyte percentage 2.8%, normal range: 6–19%). There was no history of malignancy, prior use of monoclonal antibodies or immunosuppression. This second sample was tested in three further in-house SARS-CoV-2 assays (S1 IgG capture, S1 IgM capture and hybrid N-terminal domain (S1e) DABA) and was found to be negative in all assays (BR = 0.30). The sample was also tested in an in-house pseudotype neutralisation assay and found to be negative.

## Discussion

We found considerable inter-assay variability in the relationship between anti-NP and anti-RBD IgG antibody responses in the SARS-CoV-2 post-recovery phase using the Abbott anti-NP and an in-house anti-RBD assay. This may have implications on any comparative interpretation of results across the spectrum of SARS-CoV-2 serology studies being performed as part of large-scale seroprevalence/reinfection studies, vaccine efficacy studies or, most recently, as part of clinical eligibility criteria for novel therapeutics [13,20]. Moreover, anti-RBD antibody detection using our in-house assay was significantly influenced by the severity of clinical symptomatology. The observed difference between anti-NP and anti-RBD antibody assays to detect prior cases of infection if found to be generalisable, could help optimise delayed case identification and longitudinal seroprevalence programmes in the post-vaccine era.

The majority of available commercial serological assays employed in the first year of the pandemic identified the anti-NP antibody, with many others providing unclear identification of targets [9]. Given the roll-out of large-scale vaccine programmes across Europe in 2021 and the availability of further treatment options (including combination monoclonal antibodies), anti-spike antibody assays are increasingly being used. Early studies have demonstrated reasonable specificity among different anti-NP antibody assays but limited sensitivity, particularly when applied to non-severe cases of disease [4,12,21,22]. Our data suggest that our anti-RBD DABA assay was able to detect a significantly higher proportion of cases than an anti-NP assay that had a sensitivity at the higher end of those observed. When the whole cohort was considered, it was further evident that there is two-way discordance across the assays with detection of anti-NP antibody without anti-RBD antibody and vice versa. The cause of this discordance is likely to be multi-factorial but may include differences in temporal dynamics. We observed a trend to later sampling since symptom onset and to significantly lower BR in those with discordant results compared with those with matched results. We did observe a small, but statistically significant, decline in anti-NP antibody BR with time that may further contribute to mis-matched results for those with low-level detectable responses in asymptomatic infection. The significance of this difference may become more apparent as time progresses beyond our study period. It was noted that where anti-NP antibody-positive results were matched with negative anti-RBD antibody results, the BR values were collectively close to the assay cut-off and significantly lower as a group than where results were matched (p = 0.001). Where the anti-RBD DABA has demonstrated high-fidelity performance characteristics, it is alternatively possible that the small proportion of these anti-NP antibody-positive results could represent low-level non-specific reactions or possibly result from cross-reaction with the conserved nucleocapsid of other endemic coronaviruses [23]. 

Mismatch between anti-NP and anti-RBD antibody results occurred more in older adults and it is possible that cross-reactivity is partially related to the degree of previous exposure to other coronaviruses. A small but significant reduction in anti-RBD BR with age, but not seen with anti-NP BR may alternatively reflect a response attenuated by immunosenescence in those exposed at an older age to SARS-CoV-2 but a good response to previous exposure to coronaviruses when younger. In only one PCR-confirmed case was anti-RBD not detectable, nor was there evidence of anti-NP seroconversion, and on further investigation this remained the case across four further assays including a pseudotype neutralisation assay. Advanced age may have played a role in reducing antibody levels in this case. Despite recognition of the role immunosenescence may play in the humoral response to coronaviruses [24], we did however demonstrate the presence of anti-RBD in the majority of our cohort over 65 years-old and up to 96 years-old. Furthermore, differences in assay performance, as demonstrated by comparison with PCR-positive results, may also explain a degree of this discordance. While we were able to show reasonable overall agreement between anti-NP and anti-RBD antibodies detection (87.5%), as well as a correlation between anti-NP and anti-RBD BR (ρ = 0.75), our findings suggest that presence of anti-NP antibody may not be reliably indicative of the presence of anti-RBD antibody. Future seroprevalence studies will have to account for a heterogenous landscape of prior exposure and varying vaccine uptake, and assay choices will need to be carefully considered in order to address the specific question at the time, for example as with screening individuals' eligibility for combination monoclonal antibody therapies such as Ronapreve [13]. In addition, emerging evidence in individuals with clinical resolution of robust T-cell responses in the absence of detectable antibodies further highlights the complex and multi-factorial nature of the SARS-CoV-2 immune response and potential limitations to standalone seroprevalence studies [25].

Duration of IgG antibody detection following seroconversion has not been fully characterised, although it is suggested that the strength of response may reflect disease severity and symptomology, in this study and elsewhere [4,11]. Some reports have suggested a trend of declining responses as early as 2–3 months after infection [26]. Over a similar time period, we found week-by-week variation in BR, with significant decline not occurring until after week 13 of symptoms for both anti-RBD and anti-NP. When added to the multivariable model, we observed no significant differences in anti-RBD BR relative to time but did notice a small but statistically significant decrease in anti-NP BR which would support previous observations. Of note, we did observe a significantly decreased level among asymptomatic compared with symptomatic individuals, even in the cohort with predominantly mild infection.

Availability issues may favour anti-NP antibody assays for delayed case identification programmes, yet we found that re-testing of negative samples for anti-RBD considerably increased delayed case identification rates. If further studies find our observations to be generalisable across anti-NP and anti-RBD antibody assays, programmes that employ anti-NP antibody as a screening tool may benefit from repeat testing of negative results with an anti-RBD assay. This may be particularly beneficial when following mild community infection, where a considerable proportion of people observed to be seropositive in only the anti-RBD test were asymptomatic.

At this stage it is worth noting that even when employing multiple-target assays, in rare circumstances some individuals may not show evidence of seroconversion following infection. This may have limited effect at an individual basis but where significant numbers are being surveyed in population-wide serostudies it may have considerable impact. Recent data suggests that previous infection, even in those that fail to demonstrate a detectable antibody response, correlates with a significantly reduced risk of reinfection [27]. Further investigation, including T-cell analyses, may provide valuable insight to the full spectrum of the SARS-CoV-2 immune response [25].

Our study is limited by single point measurement of SARS-CoV-2 serology and awaited longitudinal series will provide greater information around the relationship of antibody levels across time beyond our observations here. Our in-house anti-RBD assay and the Abbott anti-NP assay, while employed as one of two key initial serology assays in the UK [16], can only be considered as examples of anti-RBD and anti-NP assays. Given the different assay mechanisms it may well be that other assay types provide different results when compared. The degree of discordance may differ between assays from other manufacturers and independent cross-validation should therefore also be considered in long-term serostudies with other assays. Our multivariable model is incomplete for possible confounding factors and we were unable to account for ethnicity having an insignificant level of self-reported demographics. Likewise, this approach relies partially on participant recall of symptom onset dates which may become less accurate with longer time since onset. In addition, as those HCW with significant co-morbidities were shielding and therefore not at work to participate, across the cohort we had an insignificant representation of co-morbidities or immunosuppressed individuals to complete further analysis in this area. Similarly, it is possible that continued low-level exposure events at work may affect the window of detectable antibody in HCW and this may be relevant when considering optimal sampling times for serostudies involving non-HCW populations. Each of these factors may affect the outcome of our model if applied to wider populations. While some cases of positive anti-RBD results with negative anti-NP results could represent false positive anti-RBD results, the observation of PCR-positive cases fitting this description and a high specificity for the anti-RBD assay would suggest the contribution of anti-RBD antibody false positives to be minimal.

Observations from this study do highlight potential concerns around relying on single assays alone. Further work across a variety of anti-NP and anti-RBD assays alongside serial sampling to observe changes in antibody BR through longitudinal studies will allow further exploration of these observations.

### Conclusion

SARS-CoV-2 anti-RBD IgG antibody, using an in-house assay, showed a significant but not absolute correlation with the Abbott anti-NP antibody reactivity expressed as BR. Higher reactivity was seen in those with fever and anosmia; in contrast, cough appeared to have no effect on anti-RBD BR. Observed inter-assay variability may have implications for population-based seroprevalence and longitudinal studies. Discordance between assays appeared more apparent as time progressed from symptom onset and in those with low BR following asymptomatic infection. As anti-NP assays utilise a non-neutralising antibody, the observed discordance could limit their use for any future interpretation of anti-NP results alone if neutralising antibodies are correlated with inferred immunity. Further work comparing a range of commercial and research assays will allow for further characterisation of the dynamics associated with inter-assay variability.

## Supplementary Data

523000 Supplement
