# Supplementary material for: Variability in detection of SARS-CoV-2-specific antibody responses following mild infection: a prospective multicentre cross-sectional study, London, United Kingdom, 17 April to 17 July 2020
Source: Euro Surveill. 2022 Jan 27;27(4):2002076. doi: 10.2807/1560-7917.ES.2022.27.4.2002076 (PMC8796290; doi:10.2807/1560-7917.ES.2022.27.4.2002076)
Supplement: Supplement [file 20-02076_PALLETT_Supplement.pdf]

Supplementary information

This supplementary material is hosted by *Eurosurveillance* as supporting information alongside the article "*Variability in detection of SARS-CoV-2-specific antibody responses following mild infection: a prospective multicentre cross-sectional study, London, United Kingdom, 17 April to 17 July 2020*", on behalf of the authors, who remain responsible for the accuracy and appropriateness of the content. The same standards for ethics, copyright, attributions and permissions as for the article apply. Supplements are not edited by *Eurosurveillance* and the journal is not responsible for the maintenance of any links or email addresses provided therein.

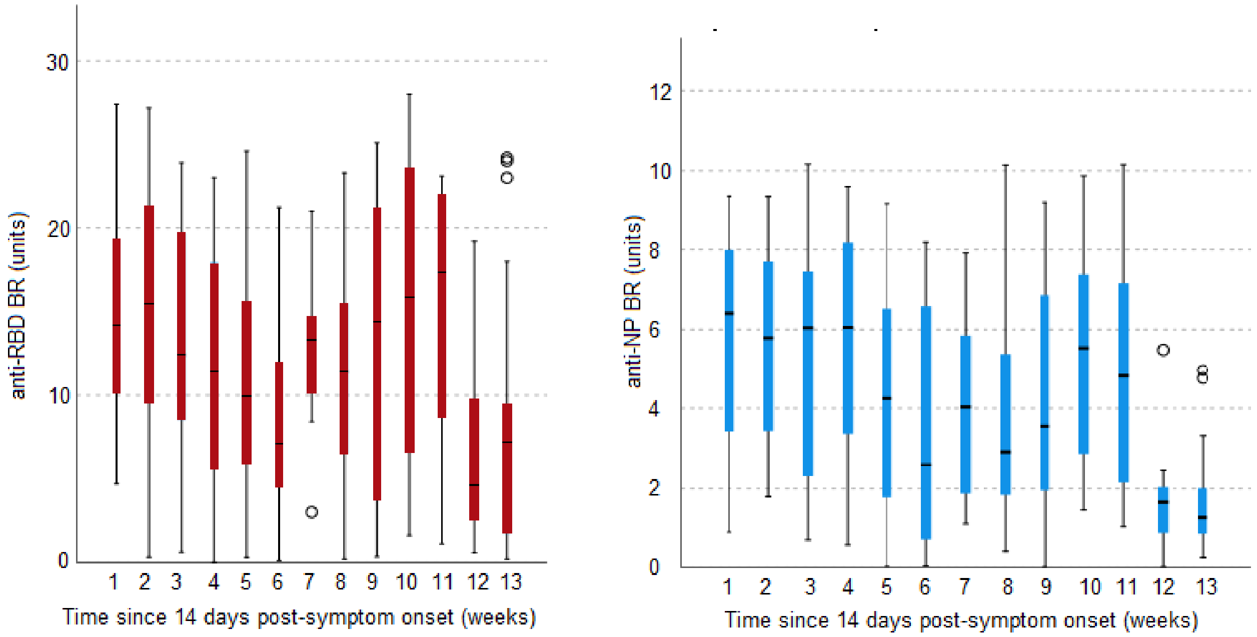

Supplementary figure 1. Binding ratio distribution of anti-RBD and anti-NP values per week beginning at 14 days post symptom onset (listed as week 1), London, United Kingdom, 17 April-17 July 2020 (n=906)**.** BR=binding ratio, anti-NP = anti-nucleocapsid antibody, anti-RBD = anti-receptor binding domain antibody. Demonstration of measured variability in individual anti-RBD and ant-NP BR values using the Kruskal-Wallis test. Week 1 therefore begins on day 15 post-symptom onset.

Dunn's post hoc analysis for anti-RBD BR

Week-week comparison	Test Statistic	Std. Error	Std. Test Statistic	Sig.	Adj. Sig.[a]
12.00-6.00	15.425	29.095	.530	.596	1.000
12.00-13.00	-16.543	27.790	-.595	.552	1.000
12.00-5.00	45.175	27.790	1.626	.104	1.000
12.00-8.00	47.075	26.024	1.809	.070	1.000
12.00-4.00	52.950	27.431	1.930	.054	1.000
12.00-9.00	62.212	25.592	2.431	.015	1.000
12.00-7.00	66.363	36.288	1.829	.067	1.000
12.00-3.00	72.055	26.024	2.769	.006	.439
12.00-2.00	84.304	24.879	3.389	<.001	.055
12.00-11.00	85.436	26.522	3.221	.001	.100
12.00-10.00	86.514	25.396	3.407	<.001	.051
12.00-1.00	86.778	23.858	3.637	<.001	.021
6.00-13.00	-1.118	29.434	-.038	.970	1.000
6.00-5.00	29.750	29.434	1.011	.312	1.000
6.00-8.00	-31.650	27.772	-1.140	.254	1.000
6.00-4.00	37.525	29.095	1.290	.197	1.000
6.00-9.00	-46.787	27.368	-1.710	.087	1.000
6.00-7.00	-50.937	37.562	-1.356	.175	1.000
6.00-3.00	56.630	27.772	2.039	.041	1.000
6.00-2.00	68.879	26.703	2.579	.010	.772
6.00-11.00	-70.011	28.239	-2.479	.013	1.000
6.00-10.00	-71.089	27.185	-2.615	.009	.696
6.00-1.00	71.353	25.753	2.771	.006	.436
13.00-5.00	28.632	28.144	1.017	.309	1.000
13.00-8.00	30.532	26.401	1.156	.248	1.000
13.00-4.00	36.407	27.790	1.310	.190	1.000
13.00-9.00	45.669	25.976	1.758	.079	1.000
13.00-7.00	49.819	36.560	1.363	.173	1.000
13.00-3.00	55.512	26.401	2.103	.036	1.000
13.00-2.00	67.761	25.274	2.681	.007	.572
13.00-11.00	68.892	26.892	2.562	.010	.812
13.00-10.00	69.971	25.783	2.714	.007	.519
13.00-1.00	70.234	24.269	2.894	.004	.297
5.00-8.00	-1.900	26.401	-.072	.943	1.000
5.00-4.00	7.775	27.790	.280	.780	1.000
5.00-9.00	-17.037	25.976	-.656	.512	1.000
5.00-7.00	-21.187	36.560	-.580	.562	1.000
5.00-3.00	26.880	26.401	1.018	.309	1.000
5.00-2.00	39.129	25.274	1.548	.122	1.000
5.00-11.00	-40.261	26.892	-1.497	.134	1.000
5.00-10.00	-41.339	25.783	-1.603	.109	1.000
5.00-1.00	41.603	24.269	1.714	.086	1.000
8.00-4.00	5.875	26.024	.226	.821	1.000
8.00-9.00	-15.137	24.077	-.629	.530	1.000
8.00-7.00	19.288	35.236	.547	.584	1.000
8.00-3.00	24.980	24.535	1.018	.309	1.000
8.00-2.00	37.229	23.318	1.597	.110	1.000
8.00-11.00	-38.361	25.063	-1.531	.126	1.000
8.00-10.00	-39.439	23.869	-1.652	.098	1.000
8.00-1.00	39.703	22.225	1.786	.074	1.000
4.00-9.00	-9.262	25.592	-.362	.717	1.000
4.00-7.00	-13.412	36.288	-.370	.712	1.000
4.00-3.00	19.105	26.024	.734	.463	1.000
4.00-2.00	31.354	24.879	1.260	.208	1.000
4.00-11.00	-32.486	26.522	-1.225	.221	1.000
4.00-10.00	-33.564	25.396	-1.322	.186	1.000
4.00-1.00	33.828	23.858	1.418	.156	1.000
9.00-7.00	4.150	34.918	.119	.905	1.000
9.00-3.00	9.843	24.077	.409	.683	1.000
9.00-2.00	22.092	22.835	.967	.333	1.000
9.00-11.00	-23.224	24.614	-.944	.345	1.000
9.00-10.00	-24.302	23.397	-1.039	.299	1.000
9.00-1.00	24.566	21.717	1.131	.258	1.000
7.00-3.00	5.693	35.236	.162	.872	1.000
7.00-2.00	17.942	34.400	.522	.602	1.000
7.00-11.00	-19.073	35.606	-.536	.592	1.000
7.00-10.00	-20.152	34.776	-.579	.562	1.000
7.00-1.00	20.415	33.668	.606	.544	1.000
3.00-2.00	12.249	23.318	.525	.599	1.000
3.00-11.00	-13.381	25.063	-.534	.593	1.000
3.00-10.00	-14.459	23.869	-.606	.545	1.000
3.00-1.00	14.723	22.225	.662	.508	1.000
2.00-11.00	-1.132	23.873	-.047	.962	1.000
2.00-10.00	-2.210	22.616	-.098	.922	1.000
2.00-1.00	2.474	20.873	.119	.906	1.000
11.00-10.00	1.078	24.411	.044	.965	1.000
11.00-1.00	1.342	22.806	.059	.953	1.000
10.00-1.00	.263	21.487	.012	.990	1.000

Each row tests the null hypothesis that the Sample 1 and Sample 2 distributions are the same.
Asymptotic significances (2-sided tests) are displayed. The significance level is .050.

a. Significance values have been adjusted by the Bonferroni correction for multiple tests.

Dunn's post hoc analysis for anti-NP BR

Week-week comparison	Test Statistic	Std. Error	Std. Test Statistic	Sig.	Adj. Sig.[a]
13.00-12.00	.955	27.790	.034	.973	1.000
13.00-6.00	55.262	29.434	1.877	.060	1.000
13.00-8.00	66.865	26.402	2.533	.011	.883
13.00-7.00	78.293	36.560	2.141	.032	1.000
13.00-5.00	85.684	28.144	3.044	.002	.182
13.00-9.00	87.809	25.976	3.380	<.001	.056
13.00-11.00	96.562	26.893	3.591	<.001	.026
13.00-10.00	120.498	25.784	4.673	<.001	.000
13.00-3.00	121.365	26.402	4.597	<.001	.000
13.00-4.00	126.305	27.790	4.545	<.001	.000
13.00-2.00	128.831	25.274	5.097	<.001	.000
13.00-1.00	135.451	24.269	5.581	<.001	.000
12.00-6.00	54.306	29.096	1.866	.062	1.000
12.00-8.00	65.910	26.024	2.533	.011	.883
12.00-7.00	77.338	36.289	2.131	.033	1.000
12.00-5.00	84.729	27.790	3.049	.002	.179
12.00-9.00	86.854	25.592	3.394	<.001	.054
12.00-11.00	95.607	26.522	3.605	<.001	.024
12.00-10.00	119.543	25.397	4.707	<.001	.000
12.00-3.00	120.410	26.024	4.627	<.001	.000
12.00-4.00	125.350	27.432	4.570	<.001	.000
12.00-2.00	127.876	24.879	5.140	<.001	.000
12.00-1.00	134.496	23.858	5.637	<.001	.000
6.00-8.00	-11.604	27.772	-.418	.676	1.000
6.00-7.00	-23.031	37.562	-.613	.540	1.000
6.00-5.00	30.423	29.434	1.034	.301	1.000
6.00-9.00	-32.547	27.368	-1.189	.234	1.000
6.00-11.00	-41.300	28.240	-1.462	.144	1.000
6.00-10.00	-65.237	27.186	-2.400	.016	1.000
6.00-3.00	66.104	27.772	2.380	.017	1.000
6.00-4.00	71.044	29.096	2.442	.015	1.000
6.00-2.00	73.570	26.703	2.755	.006	.458
6.00-1.00	80.190	25.754	3.114	.002	.144
8.00-7.00	11.428	35.236	.324	.746	1.000
8.00-5.00	18.819	26.402	.713	.476	1.000
8.00-9.00	-20.944	24.077	-.870	.384	1.000
8.00-11.00	-29.697	25.063	-1.185	.236	1.000
8.00-10.00	-53.633	23.869	-2.247	.025	1.000
8.00-3.00	54.500	24.536	2.221	.026	1.000
8.00-4.00	59.440	26.024	2.284	.022	1.000
8.00-2.00	61.966	23.318	2.657	.008	.614
8.00-1.00	68.586	22.225	3.086	.002	.158
7.00-5.00	7.391	36.560	.202	.840	1.000
7.00-9.00	-9.516	34.919	-.273	.785	1.000
7.00-11.00	-18.269	35.606	-.513	.608	1.000
7.00-10.00	-42.205	34.776	-1.214	.225	1.000
7.00-3.00	43.073	35.236	1.222	.222	1.000
7.00-4.00	48.013	36.289	1.323	.186	1.000
7.00-2.00	50.538	34.400	1.469	.142	1.000
7.00-1.00	57.159	33.668	1.698	.090	1.000
5.00-9.00	-2.125	25.976	-.082	.935	1.000
5.00-11.00	-10.878	26.893	-.404	.686	1.000
5.00-10.00	-34.814	25.784	-1.350	.177	1.000
5.00-3.00	35.681	26.402	1.351	.177	1.000
5.00-4.00	40.621	27.790	1.462	.144	1.000
5.00-2.00	43.147	25.274	1.707	.088	1.000
5.00-1.00	49.767	24.269	2.051	.040	1.000
9.00-11.00	-8.753	24.614	-.356	.722	1.000
9.00-10.00	-32.689	23.398	-1.397	.162	1.000
9.00-3.00	33.556	24.077	1.394	.163	1.000
9.00-4.00	38.496	25.592	1.504	.133	1.000
9.00-2.00	41.022	22.835	1.796	.072	1.000
9.00-1.00	47.642	21.717	2.194	.028	1.000
11.00-10.00	23.936	24.411	.981	.327	1.000
11.00-3.00	24.803	25.063	.990	.322	1.000
11.00-4.00	29.743	26.522	1.121	.262	1.000
11.00-2.00	32.269	23.873	1.352	.176	1.000
11.00-1.00	38.890	22.806	1.705	.088	1.000
10.00-3.00	.867	23.869	.036	.971	1.000
10.00-4.00	5.807	25.397	.229	.819	1.000
10.00-2.00	8.333	22.616	.368	.713	1.000
10.00-1.00	14.953	21.487	.696	.486	1.000
3.00-4.00	-4.940	26.024	-.190	.849	1.000
3.00-2.00	7.466	23.318	.320	.749	1.000
3.00-1.00	14.086	22.225	.634	.526	1.000
4.00-2.00	2.526	24.879	.102	.919	1.000
4.00-1.00	9.146	23.858	.383	.701	1.000
2.00-1.00	6.620	20.873	.317	.751	1.000

Each row tests the null hypothesis that the Sample 1 and Sample 2 distributions are the same.
Asymptotic significances (2-sided tests) are displayed. The significance level is .050.

a. Significance values have been adjusted by the Bonferroni correction for multiple tests.

Supplementary figure 2. Dunn's post hoc pairwise comparisons for week-by-week variation in binding ratio values against time since symptom onset, London, United Kingdom, 17 April-17 July 2020 (n=906)**.** BR=binding ratio, anti-NP = anti-nucleocapsid antibody, anti-RBD = anti-receptor binding domain antibody. Sig = significance**.** Week 1 therefore begins on day 15 post-symptom onset. Adjusted significance values have been calculated by use of the Bonferroni correction for multiple tests.
